# Supplementary material for: Cost-effectiveness of tuberculosis infection screening at first reception into English prisons: a model-based analysis
Source: eClinicalMedicine. 2025 May 12;83:103245. doi: 10.1016/j.eclinm.2025.103245 (PMC12140050; doi:10.1016/j.eclinm.2025.103245)
Supplement: Appendix R1 [file mmc1.pdf]

Cost-effectiveness of tuberculosis infection screening at first reception into English prisons: a model-based analysis (Appendix)

Nyashadzaishe Mafirakureva, PhD; Rachael Hunter, PhD; Claire F. Ferraro, MSc; Steve Willner; Thomas Finnie, PhD; Andrew Hayward, PhD; Andrew Lee, MD; Anjana Roy, PhD; Chantal Edge, PhD; Peter J. Dodd, PhD

1 Supplementary Methods **2**
1.1 Prison populations & flows 2
1.1.1 Model structure & equations 2
1.1.2 Available data & parametrization 4
1.1.2.1 Constraints 4
1.1.2.2 Mean duration on release 5
1.1.2.3 Priors 5
1.1.2.4 Sampling and results 6
1.2 Tuberculosis natural history and transmission 8
1.2.1 Model structure & equations 8
1.2.1.1 Equations for dynamics 9
1.2.1.2 Parameters 10
1.2.1.3 Parameter notes 14
1.2.2 Health economic outputs 14
1.3 Pathways of care for tuberculosis infection & disease 16
1.3.1 Screening new people in prison and other PPDs for tuberculosis 16
1.3.2 Latent TB infection case-finding pathway 20
1.3.3 Management of People in prison and other PPDs who develop symptoms 20
1.4 Approach to costing 20
1.4.1 Active tuberculosis disease case finding 20
1.4.2 Latent tuberculosis infection screening 22
1.4.3 Proportions/probabilities 22
1.4.4 Resource use 23
1.4.5 Unit costs 24
1.5 Analytic approach 25
1.6 Approach to modelling targeting 25
1.7 Estimated sensitivity and specificity of targeting 27
2 Supplementary Results **28**
3 References **33**

1 Supplementary Methods

1.1 Prison populations & flows

1.1.1 Model structure & equations

A model structure representing sub-populations of those in prisons and places of detention was developed after consultation focussed on what groups were important to represent because of their different accessibility to intervention and durations of exposure and retention within the prison system (see 1). These populations do not necessarily represent physically separate facilities. The aim of this model was to allow for evaluation of interventions targeted at different transfer points (not considered in this work). Within this work, it plays the role of generating a phase-type distributed dwell-time within detention.

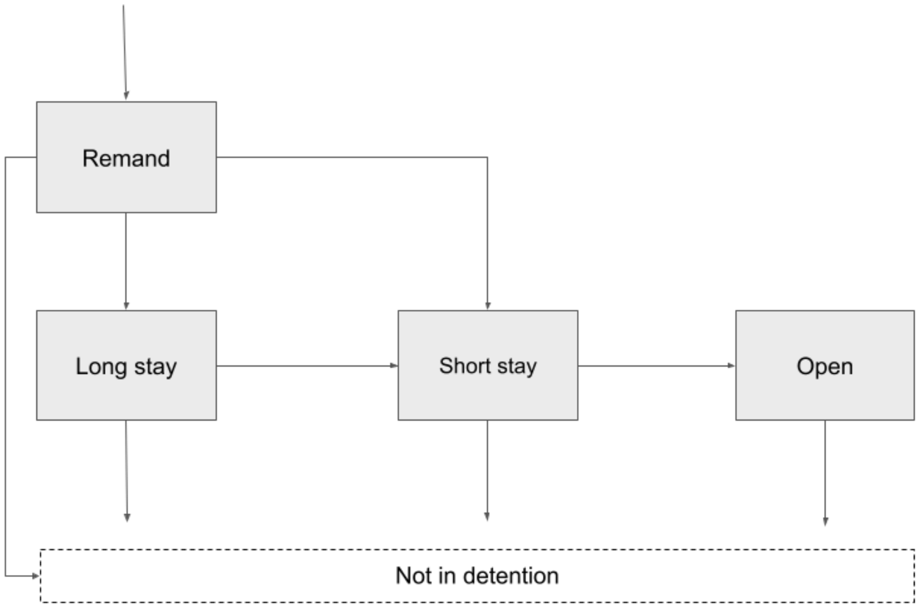

Figure A1 Model structure for detention

These subpopulations are represented as continuous counts in a continuous time compartmental model. Dynamics are represented by the following ordinary differential equations (ODEs):

$$\frac{dR}{dt} = E - \frac{R}{d_R} - \mu R$$

$$\frac{dL}{dt} = p_S p_L \frac{R}{d_R} - \tau_L L - \frac{L}{d_L} - \mu L$$
$$\frac{dS}{dt} = p_S(1 - p_L)\frac{R}{d_R} + \tau_L L - \frac{S}{d_S} - \tau_S S - \mu S$$
$$\frac{d\Omega}{dt} = \tau_S S - \frac{\Omega}{d_\Omega} - \mu\Omega$$
$$\frac{dP}{dt} = (1 - p_S)\frac{R}{d_R} + \frac{\Omega}{d_\Omega} + \frac{L}{d_L} + \frac{S}{d_S} - \mu P$$

The states and parameters in these ODEs are defined in tables 1 and 2, respectively.

Table A1 State definitions in model of detention

**Symbol**	**Meaning (stock type)**
R	Remand
L	Long stay
S	Short stay
Ω	Open
P	Released

Table A2 Parameter definitions in model of detention

**Symbol**	**Meaning**
E	Remand inflow
d_R	Remand timescale (dwell time)
d_L	Long stay timescale (dwell time)
d_S	Short stay timescale (dwell time)
d_Ω	Open timescale (dwell time)
p_S	Fraction of remand sentenced
p_L	Fraction of sentenced to long
τ_L	Transfer rate from long to short
τ_S	Transfer rate from short to open
μ	Background mortality rate

1.1.2 Available data & parametrization

In the absence of more detailed statistics to uniquely inform the detention model parameters of 2, we took the approach of using public aggregate statistics and an approximation of equilibrium to define a set of 'constraints'. We then used a Bayesian approach: these constraints were used to construct a likelihood, which we used together with our prior expectations around parameter values to generate samples from a posterior consistent with constraints.

1.1.2.1 Constraints

The constraints were used to construct a log-likelihood (LL):

$$LL \;=\; -\sum_i \frac{(C_i(p)-k_i)^2}{2(\varepsilon k_i)^2}$$

where p represents the parameters (2), and ε the proportion error tolerance, taken as 5%. This choice is intended to treat proportionate parameter errors equally. 3 shows the constraints $C_i(p)$, their values k_i, and their sources.

Table A3 Constraints and data used for flow inference

#	Calculation	Meaning	Value	Source
1	dR/dt (see above)	Remand equilibrium	0	assumed
2	dL/dt (see above)	Long equilibrium	0	assumed
3	dS/dt (see above)	Short equilibrium	0	assumed
4	$d\Omega/dt$ (see above)	Open equilibrium	0	assumed
5	$p_S \frac{R}{d_R}$	Number sentenced<br>(use sentenced & remanded)	6,193 &<br>12,747	1st reception<br>Q'ly stats
6	$N=R+L+S+\Omega$	Total population	87,489	Q'ly stats
7	$L/d_L+S/d_S+\Omega/d_\Omega$	Total releases	12,351	Q'ly stats
8	T (see below)	Mean duration at release	2.2 y	op'n data tool calc
9	Ω/N	Fraction in open	9%	Pop'n data tool calc

1.1.2.2 Mean duration on release

The mean duration at release represents time served among those released from a custodial sentence. This is the average of the mean time served from each population type:

$$T = (\frac{L}{d_L}T_L + \frac{S}{d_S}T_S + \frac{\Omega}{d_\Omega}T_\Omega) / (\frac{L}{d_L} + \frac{S}{d_S} + \frac{\Omega}{d_\Omega})$$

The mean time served on release from each type of prison must include time served while on remand, and in the case of 'short' stay population a contribution from the fraction of this population who were transferred from the 'long' stay population:

$$T_L = d_R^{-1} + d_L^{-1}$$

$$T_S = d_R^{-1} + \frac{\tau_L L}{p_S(1-p_L)R/d_R + \tau_L L} d_L^{-1} + d_S^{-1}$$

$$T_\Omega = T_S + d_\Omega^{-1}$$

1.1.2.3 Priors

The priors used for the detention model parameters are show in 4.

Table A4 Priors for the detention model parameters. N and N_+, represent normal and half-normal distributions, respectively. B represents a beta distribution; Exp represents an exponential distribution.

#	Parameter	Meaning	Value
1	p_S	Fraction of remand sentenced	B(2,2)
2	p_L	Fraction of sentenced immediately to 'long'	B(2,18)
3	d_R	Short timescale	$N_+(0.5,0.1)$ y
4	d_S	Short timescale	$N_+(1,0.5)$ y
5	d_L	Long timescale	Exp(10 y)
6	d_Ω	Open timescale	Exp(1 y)
7	τ_S	Transfer rate short -> open	Exp(0.1 y)
8	τ_L	Transfer rate long -> short	Exp(0.1 y)

1.1.2.4 Sampling and results

We used RStan to generate posterior samples using 4 chains of 2000 iterations (the first half discarded as burn-in). 5 and 2 show the results for state variables; 6 and 3 the results for parameters. Rhat convergence statistics and effective sample sizes (ESS) suggest statistical adequacy of the sample for onward use.

Table A5 Posterior state variables. CrI = credible interval. ESS=effective sample size

variable	value (95% CrI)	Rhat	ESS
N	87 300 (85 900 to 88 800)	1.00	5915
E	12 800 (12 600 to 13 000)	1.00	2734
R	15 900 (15 700 to 16 200)	1.00	4958
S	17 900 (11 600 to 25 600)	1.00	2165
L	32 600 (18 200 to 47 200)	1.01	368
Omega	15 500 (4 100 to 25 700)	1.01	333
Sentenced	6 200 (6 100 to 6 300)	1.00	5949
Released	12 300 (12 100 to 12 500)	1.00	3059

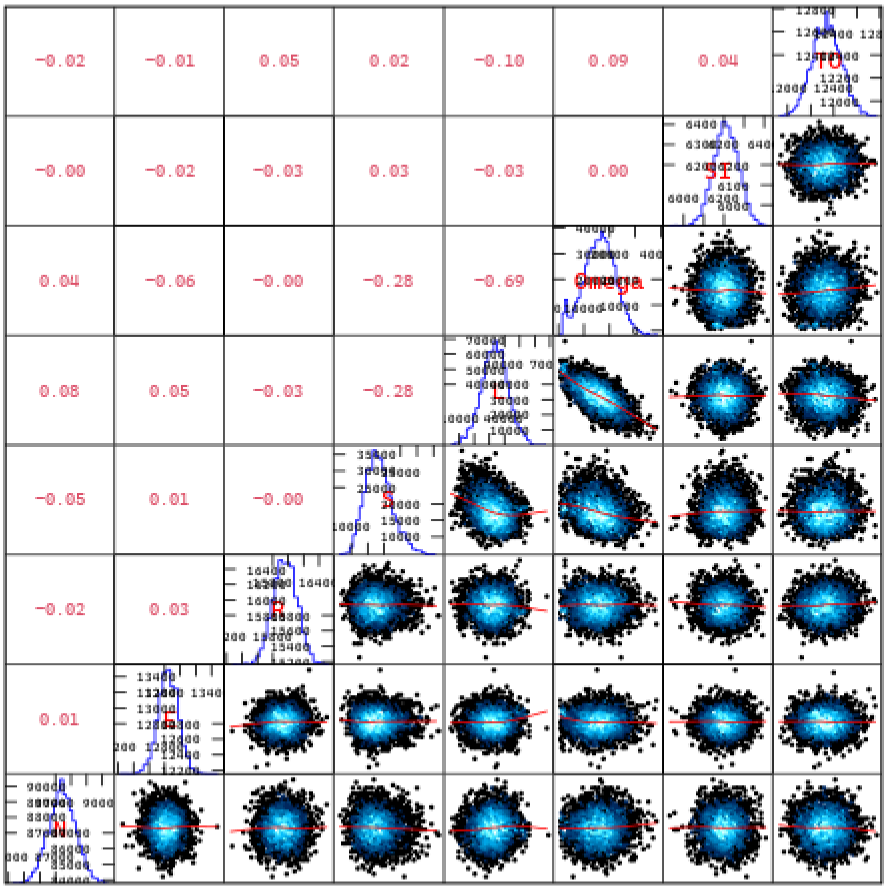

Figure A2 Corner plot for posterior state variables.

Table A6 Posterior parameter variables. CrI = credible interval. ESS=effective sample size for MCMC sampling. Rhat is a dimensionless quantity.

variable	value (95% CrI)	Rhat	ESS
dR	1.1 (1 to 1.2) years	1.00	3194
dS	2.2 (1.5 to 2.8) years	1.00	2263
dL	181 (132 to 230) years	1.01	352
$d\Omega$	5.1 (2.5 to 8.1) years	1.02	275
τS	0.094 (0.012 to 0.22) per year	1.00	1978
τL	0.056 (0.016 to 0.11) per year	1.00	2289
pS	0.52 (0.43 to 0.61)	1.00	3711
pL	0.092 (0.02 to 0.21)	1.00	4292
T	2.3 (2.1 to 2.5) per year	1.00	4746
Ω/N	0.11 (0.057 to 0.18)	1.01	648

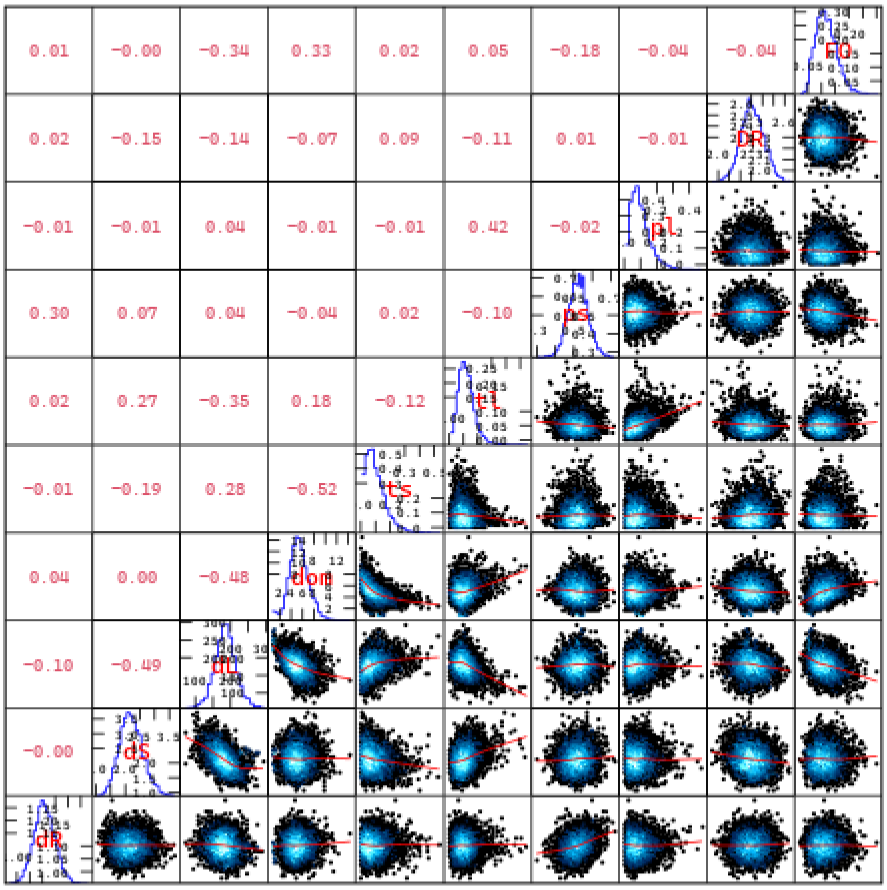

Figure A3 Corner plot for posterior parameter variables.

1.2 Tuberculosis natural history and transmission

1.2.1 Model structure & equations

The TB sector of our model (see 4 is based on standard published approaches, and also includes post-TB states to account for long-term reductions in health-related quality of life. Not shown on this diagram are 3 TPT-related strata: never had TPT; currently protected by TPT; previous TPT (no longer protected). To be consistent with long-lasting TPT protection for HIV-uninfected people observed in low- to moderate-incidence settings,[1] we consider that protection from TPT has a average duration of $d_{tpt} = 20$ years.

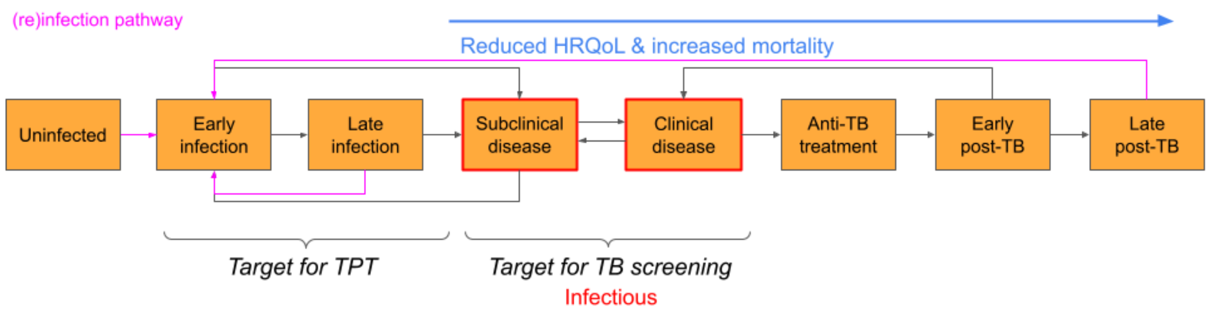

Figure A4 Structure of TB model. Purple flows are driven by force-of-infection. Red outlined boxes are infectious. All arrows shown have only one head.

1.2.1.1 Equations for dynamics

The full ordinary differential equations (ODEs) describing the detention model, TB dynamics, and TPT are described below. We used two indices:
$i = 1, \cdots, 5$ represents the prison population layer: (1=remand, 2=short, 3=long, 4=open, 5=released)
$j = 1, \cdots, 3$ represents the TPT state: (1=never had, 2=on TPT, 3=previous TPT)

Table A7 Key to state/compartment names in equations

Compartment name above	Uninfected	Early infection	Late infection	Subclinical disease	Clinical disease	Anti-TB treatment	Early post-TB	Late post-TB
ODE symbol below	U	E	L	SD	CD	ATT	PE	PL

Letters $U,\ E,\ L,\ SD,\ CD,\ ATT,\ PE,\ PL$ represent the states in the figure from left to right. U = Uninfected; E = Early infection; L = Late infection; SD = Subclinical Disease; CD = Clinical Disease; ATT = Anti-TB Treatment; PE = Post-TB (Early); PL = Post-TB (Late7 Table A7).

$$\frac{dU_{ij}}{dt} = -\lambda_i U_{ij} - \mu U_{ij} + I \times f_{ij}^{U} + M_{ij}^{U}$$

$$\frac{dE_{ij}}{dt} = \lambda_i (U_{ij} + v[L_{ij} + PE_{ij} + PL_{ij}]) - (\pi + \sigma)E_{ij} - \mu E_{ij} + I \times f_{ij}^{E} + M_{ij}^{E} + T_{ij}^{E}$$

$$\frac{dL_{ij}}{dt} = \sigma E_{ij} + (1 - \phi)\frac{CD_{ij}}{d} - v\lambda_i L_{ij} - \epsilon L_{ij} - \mu L_{ij} + I \times f_{ij}^{L} + M_{ij}^{L} + T_{ij}^{L}$$

$$\frac{dSD_{ij}}{dt} = \pi E_{ij} + \epsilon L_{ij} - wSD_{ij} - \mu SD_{ij} + I \times f_{ij}^{SD} + M_{ij}^{SD}$$

$$\frac{dCD_{ij}}{dt} = wSD_{ij} + \rho PE_{ij} - \mu CD_{ij} + I \times f_{ij}^{CD} + M_{ij}^{CD} - \frac{CD_{ij}}{(1-CDR)d}$$

$$\frac{dATT_{ij}}{dt} = \frac{CDR \times CD_{ij}}{(1-CDR)d} - \frac{ATT_{ij}}{l} - \mu ATT_{ij} + I \times f_{ij}^{ATT} + M_{ij}^{ATT}$$

$$\frac{dPE_{ij}}{dt} = (1 - \psi)\frac{ATT_{ij}}{l} - \rho PE_{ij} - H\mu PE_{ij} - \lambda_i vPE_{ij} - PE_{ij}/l_P + I \times f_{ij}^{PE} + T_{ij}^{PE}$$

$$\frac{dPL_{ij}}{dt} = PE_{ij}/l_P - H\mu PL_{ij} - \lambda_i v PL_{ij} + I \times f_{ij}^{PL} + M_{ij}^{PL} + T_{ij}^{PL}$$

Definitions and parameter sources are described in the next section and 8.

1.2.1.2 Parameters

We assume that both clinical and subclinical disease contribute equally to transmission, in the absence of evidence for differential infectiousness.[2] Consequences of transmission one released from detention are handled via multiplier approximation (see below, Health economic outputs).

$$\lambda_i = (1 - \delta_{i5})\beta \frac{\sum_{ij}(SD_{ij} + CD_{ij})}{\sum_{i=1}^{4} N_{ij}}$$

The total population in each prison population i and TPT stratum j is given by:

$$N_{ij} = U_{ij} + E_{ij} + L_{ij} + SD_{ij} + CD_{ij} + ATT_{ij} + PE_{ij} + PL_{ij}$$

M_{ij}^{X} implements the flows between the prison populations (changes in i) for each disease state X, defined by the ODEs in the first section. That is:

$$M^X = \begin{bmatrix} -d_R^{-1} - \mu & 0 & 0 & 0 & 0 \\ p_S(1 - p_L)/d_R & -d_S^{-1} - \tau_S - \mu & \tau_L & 0 & 0 \\ p_S p_L/d_R & 0 & -d_L^{-1} - \tau_L - \mu & 0 & 0 \\ 0 & \tau_S & 0 & -d_\Omega^{-1} - \mu & 0 \\ 0 & 0 & 0 & d_\Omega^{-1} & -\mu \end{bmatrix}$$

T_{ij}^{X} implements the flows between the TPT layers (changes in j) for each relevant disease state X. This amounts to an ageing out of TPT protection over a timescale T_p. Inflows to TPT (j=2) occur at entry to the detention system, that is: $T_{ij}^{X} = \left(\delta_{j3} - \delta_{j,2}\right)X_{i2}/d_{tpt}$.

I represents the remand inflow and f_{ij}^{X} represents the fraction of the inflow in state X_{ij} (for $j < 5$).

The inflow fractions f_{ij}^{X} are defined by:

$$f_{ij}^{U} = \delta_{i1}\delta_{j1}f^{U}$$
$$f_{ij}^{E} = \delta_{i1}[(1 - tpt)\delta_{j1} + tpt\,\delta_{j2}]f^{E}$$
$$f_{ij}^{L} = \delta_{i1}[(1 - tpt)\delta_{j1} + tpt\,\delta_{j2}]f^{L}$$
$$f_{ij}^{SD} = \delta_{i1}\delta_{j1}[1 - att]f^{SD}$$
$$f_{ij}^{CD} = \delta_{i1}\delta_{j1}[1 - att]f^{CD}$$
$$f_{ij}^{ATT} = \delta_{i1}\delta_{j1}[f^{ATT} + att\,(f^{SD} + f^{CD})]$$
$$f_{ij}^{PE} = \delta_{i1}[(1 - tpt)\delta_{j1} + tpt\,\delta_{j2}]f^{PE}$$
$$f_{ij}^{PL} = \delta_{i1}[(1 - tpt)\delta_{j1} + tpt\,\delta_{j2}]f^{PL}$$

where δ_{ij} is the Kronecker delta, and *tpt* and *att* represent the coverage of TPT and ATT initiation among eligible populations at entry. The fractions f^{X} represent the fractions of the newly detained population in the TB state *X*.

The initial state for the ODEs is defined by a multiplicative model:

$$X_{ij}(t = 0) = f_{0}^{X} f_{i}^{det} f_{j}^{tpt}.$$

The detection initial fractions, f_{i}^{det}, are defined by samples from the values in 5

The TPT initial fractions, $f_{i}^{tpt} = \delta_{i1}$, are defined to imply everyone is TPT naive.

Finally, the TB initial fractions are varied during the probabilistic sensitivity analysis (see Analytic approach, below) according to a heuristic parametrized by a force of infection λ, given a timescale parameter $T = 20$ years.

$$f_{0}^{U} = e^{-\lambda T}$$
$$f_{0}^{E} = (1 - e^{-\lambda T})(2\lambda)/\Delta$$
$$f_{0}^{L} = (1 - e^{-\lambda T})(0.1)/\Delta$$
$$f_{0}^{SD} = (1 - e^{-\lambda T})(\lambda/30)/\Delta$$
$$f_{0}^{CD} = (1 - e^{-\lambda T})(\lambda/30)/\Delta$$
$$f_{0}^{ATT} = (1 - e^{-\lambda T})(\lambda/30)/\Delta$$
$$f_{0}^{PE} = (1 - e^{-\lambda T})(\lambda/15)/\Delta$$
$$f_{0}^{PL} = (1 - e^{-\lambda T})(2\lambda/15)/\Delta$$

with denominator $\Delta = 2.3\lambda + 0.1$.

Table A8 TB model parameters.

HR= hazard ratio; CFR= case fatality ratio; ATT=anti-TB treatment; TPT=tuberculosis preventive therapy; TBI=TB infection; B=beta distribution; LN=log-normal distribution.

Symbol	Meaning	Distribution	value	Source
CDR	Case detection ratio	B(41.80,5.22)	89% (79%-96%)	[i] Inventory survey[3]
λ_0	Initial force-of-infection per year	LN(log(0.01),0.75)	0.01 (0.00-0.04)	Assumption
ϕ	CFR untreated TB (over background mortality)	B(25.48,33.78)	43% (31%-56%)	WHO analysis[4] of Tiemersma[5]
ψ	CFR treated TB	B(162,3264)	4.7% (4.0-5.5%)	[ii] England data[6]
d	Duration untreated TB (years)	LN(1.1,0.2)	3.0 (2.0-4.4)	[iii]Tiemersma[5]
l	Duration ATT (years)		0.5	Typical DS-TB regimen duration
l_P	Duration of early post-TB (years)		2	Definition
σ	Stabilisation rate per year	LN(0.62,0.068)	1.86 (1.62-2.12)	Ragonnet[7]
π	Fast progression rate per year	LN(-2.837,0.32)	0.06 (0.03-0.11)	Ragonnet[7]
ϵ	Slow progression rate per year	LN(-6.89,0.58)	0.001 (0.0-0.003)	Ragonnet[7]
v	TBI protection, HR	B(20.7,77.9)	0.23 (0.14-0.30)	Andrews[8]
H	Causal post-TB mortality HR	LN(0.131,0.072)	1.14 (0.99-1.31)	[iv]Menzies[9]
μ	Background mortality rate per year		1/50	Assumption
ρ	Relapse rate per year	LN(-3.95,0.27)	0.02 (0.01-0.03)	Crampin[10]
w	Symptom progression timescale per year	LN(-0.693,0.970)	0.50 (0.07-3.35)	[v]Frascella[11]
HR_{tpt}	TPT protection (HR) in TBI+	LN(-1.772,0.089)	0.17 (0.14-0.20)	Martinez[12]
d_{tpt}	TPT protection duration (years)		20	[vi]Salazar-Austin [1]

R-1	Outside transmission multiplier	LN(log(0.94),0.1)	0.94 (0.77-1.14)	[vii]England data[6]

1.2.1.3 Parameter notes

(see 8 source column)

i) As interpreted by WHO based on the inventory study of Anderson et al.[3]
ii) Assuming same mortality among those lost to follow-up.
iii) Assumed variance
iv) While Romanowski et al.[13] find a higher HR, this is not all causally due to TB. Menzies et al[9] find a range of country values with a heuristic approach used here. Romanowski et al[14] found a ~30% increase in hospitalisation after TB in an interrupted time-series analysis
v) Around half of prevalent TB subclinical, and typical global duration of TB ~ 1 year
vi) Decadal duration of protection observed in low- and medium-TB transmission settings
vii) Decline of 3%/year + generation time of 2 years

1.2.2 Health economic outputs

To calculate $X(t)$, the discounted cumulative value of a rate $x(t)$ from the intervention time T_{int}, i.e.

$$X(T) = \int_{T_{int}}^{T} dt\, x(t)\, e^{-r(t-T_{int})}$$

We introduced auxiliary differential equations:
$X(0) = 0$
$X'(t) = x(t)\, e^{-r(t-T_{int})} 1(t > T_{int})$

In particular, cumulative costs were the integral over

$$c(t) = \left(U_{screen}F_{screen} + U_{tpt}F_{tpt} + U_{attscr}F_{att}\right) \times I + \frac{CDR}{(1-CDR)d} \times \left(U_{attin}\sum_{i=1,j}^{4} CD_{i,j} + R \times U_{attout}\sum_{j} CD_{5,j}\right)$$

The first component being the fraction of inflow resulting in TPT and ATT times by the relevant unit costs, the second component representing the treatment costs for those passively detected within or outside the prison system.

The factor R is a multiplier that accounts for indirect contributions to TB incidence, mortality, and costs due to transmission in the non-detained population. This represents a next-generation approximation to explicit modelling of nonlinear transmission in the non-detained population (reasonable due to relatively low per capita incidence). The $R - 1$ secondary cases in the community are approximated as occurring at the same time. We choose a value of R by reference to the declining

community rate of TB notifications in England and an assumed generation time (see above). Implicitly, secondary cases are assumed to have the same average outcomes as primary cases.

The decrement in quality of life is driven by the rate

$$q(t) = \left(\Delta_{tbd} \times \sum_{ij} CD_{ij} + \Delta_{ptb} \times \sum_{ij}(PE_{ij} + PL_{ij})\right) + (R - 1)\left(\Delta_{tbd} \times \sum_{j} CD_{5j} + \Delta_{ptb} \times \sum_{j}(PE_{5j} + PL_{5j})\right)$$

The total TB mortality rate is

$$m(t) = \frac{\phi}{d}\sum_{ij} CD_{ij} + \frac{\psi}{l}\sum_{ij} ATT_{ij} + (H - 1)\mu\sum_{ij}(PE_{ij} + PL_{ij}) + \\ (R - 1) \times \left(\frac{\phi}{d}\sum_{j} CD_{5j} + \frac{\psi}{l}\sum_{j} ATT_{5j} + (H - 1)\mu\sum_{j}(PE_{5j} + PL_{5j})\right)$$

The discounted life-years lost due to TB are calculated as

$$LYL(T) = \int_{T_{int}}^{T} dt\, m(t)e^{-r(t-T_{int})}\left(1 - e^{-r\,LE}\right)$$

That is accruing life years for a life lost at time t as $\int_{t}^{t+LE} dt\, e^{-rt}$: the sum of discounted years through to death.

The HRQoL decrement during TB disease is taken as Δ_{tbd}= 0.333 (0.224 – 0.454) B(21.152,42.367), based on GBD estimates. The post-TB HRQoL decrement is based on the analysis in Menzies et al.[9] which found Δ_{ptb} had a median of 0.036 and a range of 0.006 to 0.088, which was used to parametrize a log-normal distribution LN(-3.324,0.486).

1.3 Pathways of care for tuberculosis infection & disease

Care pathways for the diagnosis, management, and prevention of tuberculosis in prisons and places of detention were established by reviewing relevant documents related to current practices and guidelines. These documents include a) NICE guideline NG33 on Tuberculosis: clinical diagnosis and management of tuberculosis, and measures for its prevention and control; and b) UKHSA's draft guidance on Active and Latent Case finding and management of Tuberculosis in prisons, immigration removal centres and other prescribed places of detention in England. Three separate but linked pathways were identified: 1) Screening new people in prison and other PPDs for tuberculosis; 2) Latent TB infection case-finding pathway; and, 3) Management of People in prison and other PPDs who develop symptoms. The developed diagrammatic conceptual models of patient pathways of care were discussed and agreed upon with relevant stakeholders (UKHSA and PPDs).

1.3.1 Screening new people in prison and other PPDs for tuberculosis

This pathway describes screening new people in PPDs for active tuberculosis disease. This includes tuberculosis risk assessment, tuberculosis investigations for people with symptoms, anti-tuberculosis treatment initiation for those diagnosed with tuberculosis disease and contact investigation (incident or outbreak management). The initial tuberculosis risk assessment should be done within 48 hours of arrival by verbal symptom screening and can include a chest x-ray where facilities exist. Patients with positive verbal screening and/or chest x-ray results are evaluated for active tuberculosis disease. Evaluation for active disease includes PPD doctor/GP assessment, laboratory investigations (typically

Screening new people in prison and other PPDs for TB

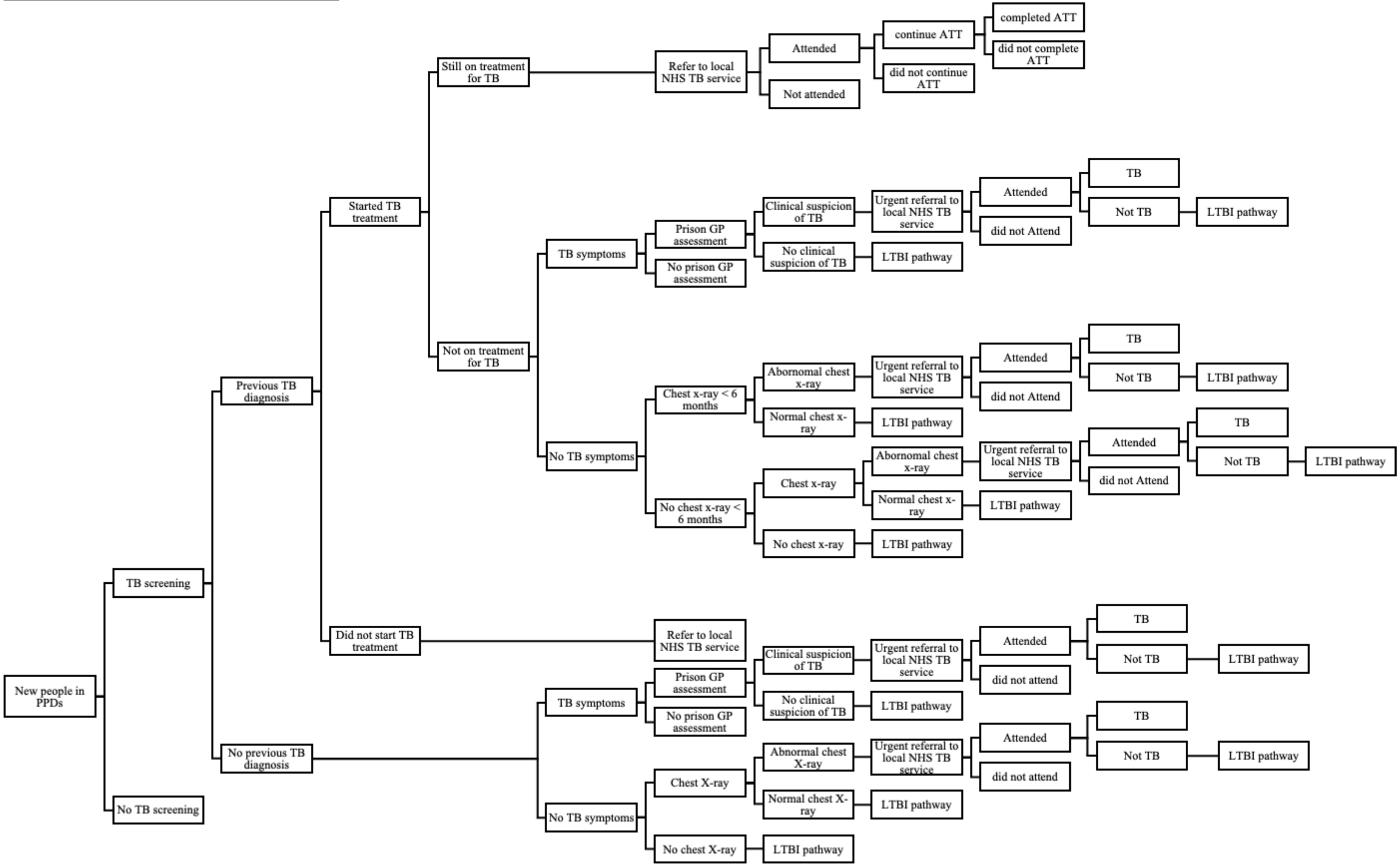

Latent TB infection case-finding pathway

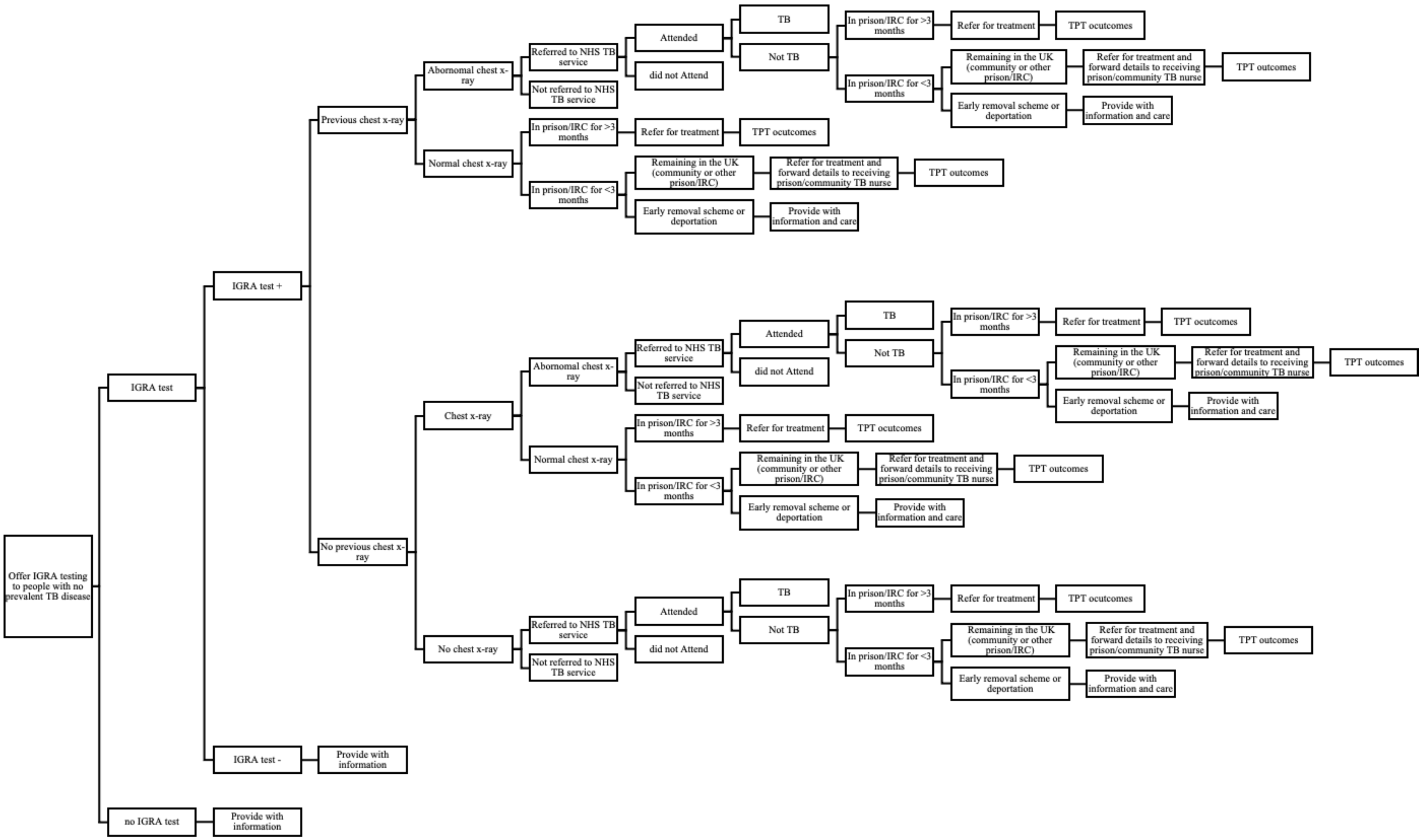

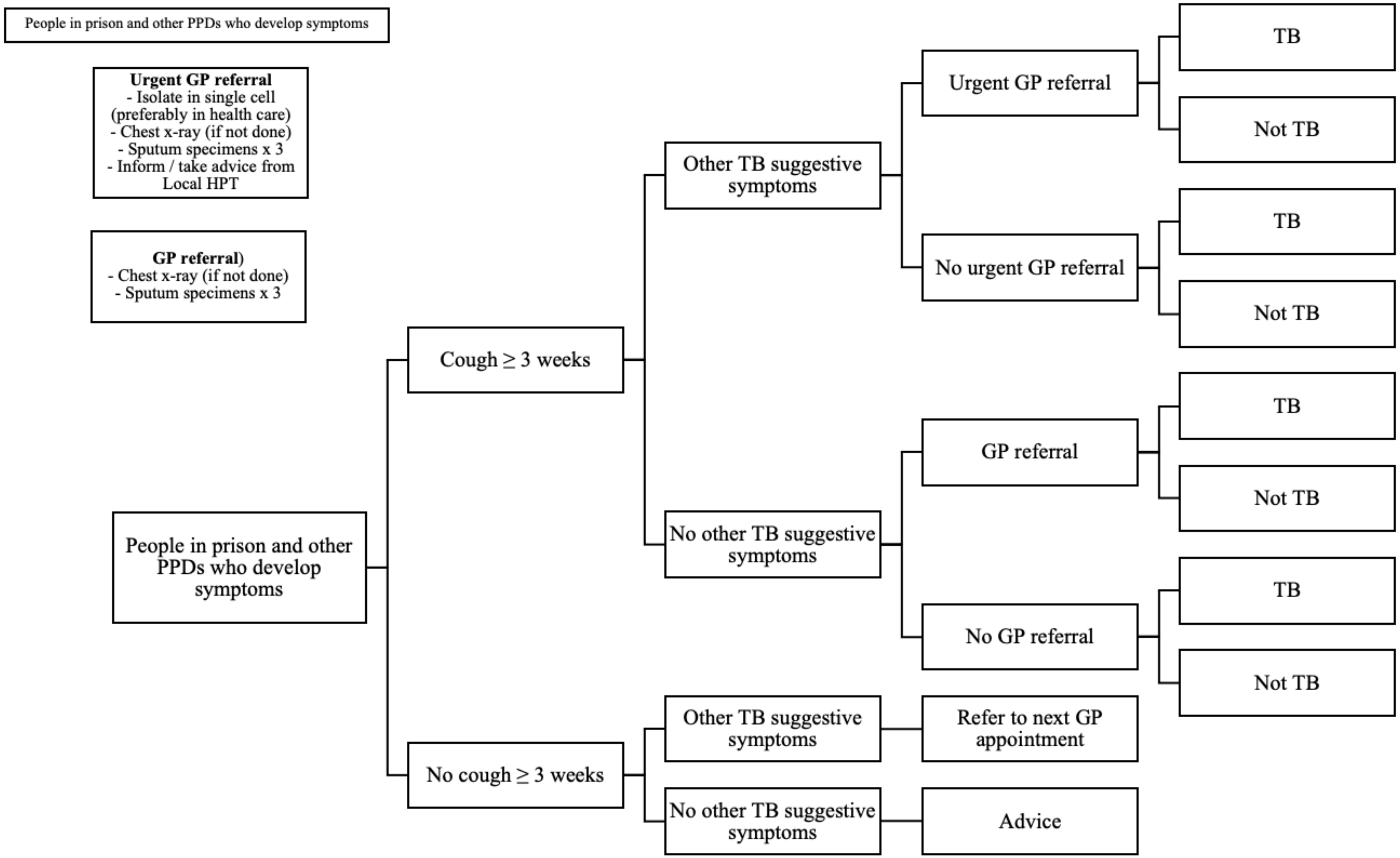
People in prison and other PPDs who develop symptoms
Urgent GP referral
- Isolate in single cell (preferably in health care)
- Chest x-ray (if not done)
- Sputum specimens x 3
- Inform / take advice from Local HPT
GP referral)
- Chest x-ray (if not done)
- Sputum specimens x 3
People in prison and other PPDs who develop symptoms
Cough ≥ 3 weeks
No cough ≥ 3 weeks
Other TB suggestive symptoms
No other TB suggestive symptoms
Other TB suggestive symptoms
No other TB suggestive symptoms
Urgent GP referral
No urgent GP referral
GP referral
No GP referral
Refer to next GP appointment
Advice
TB
Not TB
TB
Not TB
TB
Not TB
TB
Not TB

tuberculosis microscopy and culture on sputum samples) and patient referral to the local NHS TB service for further investigations. Patients in PPDs with confirmed pulmonary tuberculosis are treated using directly observed therapy (DOT) and managed by the local NHS TB service. Contact tracing is initiated for all cases of smear-positive pulmonary tuberculosis. Screening for latent tuberculosis (LTBI) is recommended for patients with verbal screening and/or chest x-ray results suggesting that active TB disease is unlikely.

1.3.2 Latent TB infection case-finding pathway

This is a follow-on pathway for LTBI screening when active tuberculosis disease has been excluded. The pathway includes testing all individuals meeting the NICE-based eligibility criteria for LTBI using the IGRA test, using chest X-ray as a rule-out test for active tuberculosis for patients with a positive IGRA test and providing DOT-based LTBI treatment for those who are eligible.

1.3.3 Management of People in prison and other PPDs who develop symptoms

Patients in prisons presenting with symptoms suggestive of active tuberculosis disease undergo PPD doctor/GP assessment, laboratory investigations (typically tuberculosis microscopy and culture on sputum samples) and patient referral to the local NHS TB service for further investigations. Those diagnosed are initiated on appropriate treatment and contact tracing is initiated as indicated.

1.4 Approach to costing

The established care pathways were used as the basis for costing. The cost analysis primarily used a standard health system perspective following the NICE reference case but identified and included HMPPS costs relevant to implementing prison-based healthcare interventions. Resources required for activities along the care pathways were identified and the quantities of the resources estimated based on guidelines or literature. Resource use was valued by multiplying the resource use and relevant unit costs. Unit costs were obtained from relevant sources such as NHS reference costs, PSSRU unit costs, PPD pilot projects and literature. Costs that accrue for each care pathway were calculated by summing up the estimated costs for each resource item. Resource use and costs for the main activities in each pathway are described below.

1.4.1 Active tuberculosis disease case finding

Table A9 Active tuberculosis disease case finding resource use parametrization

**Activity/step**	**Resource (s)**	**Cost (s)**	**Tree parameter (s)**[1]	**NHS/PPD**
TB symptom screening	Prison/PPD nurse time	cost.tb.sympt.screen	cost.tb.sympt.screen + cost.overheads + cost.prisoner.incentive	PPD
	Space & materials	cost.overheads		PPD
NHS TB services referral for anti-TB treatment	Single-cell or healthcare unit isolation	cost.prison.cell.isolation	**pIsolation***cost.prison.cell.isolation	PPD
Attending NHS TB services referral for anti-TB treatment	Outpatient visit	cost.nhs.tb.service	cost.nhs.tb.service + cost.prison.escort	NHS
	Prison security & transport	cost.prison.escort		PPD

Prison GP assessment	Single-cell or healthcare unit isolation	cost.prison.cell.isolation	**pIsolation***cost.prison.cell.isolation + cost.prison.gp.assess	PPD
	Prison GP time	cost.prison.gp.assess		PPD
Chest X-ray[2]	Chest x-ray	cost.chest.xray	cost.chest.xray	NHS
	Prison security & transport	cost.prison.escort	cost.prison.escort	PPD
Attending NHS TB services referral for TB diagnosis	Outpatient visit	cost.nhs.tb.service	cost.nhs.tb.service + cost.prison.escort + cost.tb.diagnosis	NHS
	Prison security & transport	cost.prison.escort		PPD
	TB investigations	cost.tb.investigations		NHS
TB diagnosis & contact tracing	Single-cell or healthcare unit isolation	cost.prison.cell.isolation	**pIsolation***cost.prison.cell.isolation + **nContacts***cost.contact.tracing	PPD
	Cost per person contact-traced	cost.contact.tracing		NHS/PPD
Anti-TB treatment medicines	DSTB medicines	cost.dsatt.drugs	**pDSTB*durDSTB***cost.dsatt.drugs + (1-**pDSTB**)***durMDRTB***cost.mdratt.drugs	NHS
	MDR-TB medicines	cost.mdratt.drugs		NHS
Directly observed therapy (DOT)	Prison/PPD officer or nurse time	cost.att.dots	cost.att.dots*(**pDSTB*durDSTB** + (1-**pDSTB**)***durMRDTB**)	PPD
TB inpatient stay	Standard isolation	cost.dstb.ipd	**pDSTB*smear.positive*DurDSTBIsolation*(cost.dstb.ipd + cost.prison.bedwatch) + (1-pDSTB)*smear.positive*DurMDRTBIsolation*(cost.mdrtb.ipd.smear.positive + cost.prison.bedwatch)**	NHS
	Prison security & transport	cost.prison.bedwatch		PPD
	Negative-pressure isolation cost	cost.mdrtb.ipd.smear.positive		NHS
	Prison security	cost.prison.bedwatch		PPD
	Non-isolation inpatient cost	cost.mdrtb.ipd.smear.negative		NHS
	Prison security & transport	cost.prison.escort		PPD
TB outpatient visit (within 5 days and then monthly)*	NHS TB service nurse time	cost.dstb.opd.visit	**pDSTB*dstb.visits***(cost.dstb.opd.visits + cost.prison.escort) + (1-**pDSTB**)***mdrtb.visits***(cost.mdrtb.opd.visits + cost.prison.escort)	NHS
	Prison security & transport	cost.prison.escort		PPD
	NHS TB service nurse time	cost.mdrtb.opd.visit		NHS
	Prison security & transport	cost.prison.escort		PPD
**Notes**<br>[1]Some cost parameters are not explicitly included on the trees and will have to be entered as formulae. [1]Parameters in bold are not explicitly included on the tree but may be required to compute costs.<br>[2]Chest X-ray cost may include Prison security & transport costs if done outside the prison				

1.4.2 Latent tuberculosis infection screening

Table A10 Latent tuberculosis infection screening resource use parametrization

Activity/step	Resource (s)	Cost (s)	Tree parameter (s)¹	NHS/PPD
IGRA test	IGRA test	cost.igra.test	cost.igra.test	NHS
Chest X-ray*	Chest x-ray	cost.chest.xray	cost.chest.xray	NHS
	Prison security & transport	cost.prison.escort	cost.prison.escort	PPD
NHS TB services referral for TB investigations	Single-cell or healthcare unit isolation	cost.prison.cell.isolation	**pIsolation***cost.prison.cell.isolation	PPD
Attending NHS TB services referral for TB diagnosis	Outpatient visit	cost.nhs.tb.service	cost.nhs.tb.service + cost.prison.escort + cost.tb.diagnosis	NHS
	Prison security & transport	cost.prison.escort		PPD
	TB investigations	cost.tb.investigations		NHS
Attending NHS TB services referral for LTBI treatment	Outpatient visit	cost.nhs.tb.service	cost.nhs.tb.service + cost.prison.escort	NHS
	Prison security & transport	cost.prison.escort		PPD
LTBI treatment medicines	LTBI medicines	cost.ltbi.drugs	**durTPT** *cost.ltbi.drugs	NHS
Directly observed therapy (DOT)	Prison/PPD officer or nurse time	cost.tpt.dots	**durTPT** *cost.tppt.dots	PPD
LTBI outpatient visit (within 5 days and then monthly)*	NHS TB service nurse time	cost.tpt.opd.visits	**ltbi.visits***(cost.tpt.opd.visits + cost.prison.escort)	NHS
	Prison security & transport	cost.prison.escort		PPD

Notes
*Chest X-ray examinations were assumed to be performed in the NHS and included x-ray cost in addition to prison security & transport costs

1.4.3 Proportions/probabilities

Table A11 Proportions and probabilities in care cascades

Name	Description	Distribution	Estimate (Range)	Source
ltbi.prev	Prevalence of LTBI in residents	B(1.90679,10.80514)	0.148 (0.073 - 0.20)	Cords et al. 2021[15]
tb.sympt.screen	Verbal TB symptom screening within 48 hours of arrival	B(30.21696,3.35744)	0.90 (0.87 - 0.93)	Calculated using data from prison pilots
previous.tb.started.att	Past TB diagnosis, started anti-TB treatment	B(30.21696,3.35744)	0.90 (0.87 - 0.93)	Assumption
prev.tb.continue.att	Attending NHS TB services referral & continue anti-TB treatment	B(2.85744,1.90496)	0.60 (0.46 - 0.76)	Assumption
prev.tb.still.on.att	Past TB diagnosis, started & still on anti-TB treatment	B(2.85744,1.90496)	0.60 (0.46 - 0.76)	Assumption
sens.symptom	Sensitivity for any TB symptom (cough, haemoptysis, fever, night sweats, weight loss) for screening for TB disease	B(77.02006,31.4589)	0.71 (0.68 - 0.74)	WHO consolidated guidelines on tuberculosis, 2021 [16]
spec.symptom	Specificity for any TB symptom (cough, haemoptysis, fever, night sweats, weight loss) for screening for TB disease	B(46.17545,25.97369)	0.36 (0.32 - 0.40)	WHO consolidated guidelines on tuberculosis, 2021 [16]

sens.any.ab n.xray	Sensitivity of chest radiography (any abnormality) for screening for TB disease	B(508.2257,3 2.43994)	0.94 (0.93 - 0.95)	WHO consolidated guidelines on tuberculosis, 2021 [16]
spec.any.ab n.xray	Specificty of chest radiography (any abnormality) for screening for TB disease	B(272.3528,3 3.66158)	0.11 (0.09 - 0.12)	WHO consolidated guidelines on tuberculosis, 2021 [16]
sens.xpert	Sensitivity of Xpert MTB/RIF Utra for pulmonary TB in adults	B(159.0112,1 5.91862)	0.91 (0.897 - 0.925)	Zifodya et al. 2021 [17]
spec.xpert	Specificty of Xpert MTB/RIF Utra for pulmonary TB in adults	B(318.2237,1 4.64628)	0.956 (0.949 - 0.963)	Zifodya et al. 2021 [17]
gp.assessme nt	Past TB diagnosis, started & completed anti-TB treatment, with TB symptoms, Prison GP assessment	B(30.21696,3 .35744)	0.80 (0.73 - 0.90)	Assumption
clinical.tb.s uspicion	Clinical TB suspicion	B(54.41904,6 .04656)	0.928 (0.877 - 0.928)	Assumption
xray	Proportion of residents receiving a chest xray	B(19.69357,5 .882496)	0.77 (0.56 - 0.88)	Calculated using data from prison pilots
attend.nhs.r eferral	Proportion attending NHS TB services referral	B(7.6673,2.3 715)	0.76 (0.70 - 0.86)	Davies et al. 2020[18]
igra.tested	Received IGRA test	B(132.8967,7 8.05047)	0.63 (0.61 - 0.65)	Calculated using data from prison pilots
igra.test.pos itive	Positive IGRA test	B(34.57146,1 95.9049)	0.15 (0.14 - 0.17)	Calculated using data from prison pilots
att.initiation	Starting anti-tuberculosis treatment	B(7.067597,1 .766899)	0.80 (0.71 - 0.90)	TB treatment and outcomes, England, 2021 [19]
completing. att	Completing anti-tuberculosis treatment	B(7.067597,1 .766899)	0.80 (0.71 - 0.90)	TB treatment and outcomes, England, 2021 [19]
starting.tpt	Starting tuberculosis preventive therapy	B(7.067597,1 .766899)	0.80 (0.71 - 0.90)	TB treatment and outcomes, England, 2021 [19]
completing. tpt	Completing tuberculosis preventive therapy	B(7.067597,1 .766899)	0.80 (0.71 - 0.90)	TB treatment and outcomes, England, 2021 [19]
pIsolation	Proportion of prisoners isolated in single cells	B(1.012551,3 9.4895)	0.025 (0.007 - 0.034)	Assumption
prev.tb.dx	Past TB diagnosis	Fixed	0.02	Calculated using data from prison pilots
prev.xray	Previous chest X-ray < 6 months	Fixed	0.01	Calculated using data from prison pilots
abnormal.xr ay	Abnormal chest X-ray	Fixed	0.06	Calculated using data from prison pilots
staying.o3. months	Staying in prison/ICRC > 3 months	Fixed	0.79	Calculated using data from prison pilots
staying.u3. months.uk	Stay in prison/ICRC <3 months, remaining in the UK	Fixed	0.79	Assuming same figures as above

1.4.4 Resource use

Table A12 Other resource use parameters

Name	Description	Units	Distribution	Estimate (Range)	Source
nContacts	Number of contacts examined per primary case	Contacts per case	LN(1.837889,0.2604345)	6.3 (5.9-6.7)	Pareek et al. 2013 [20]
verbal_screen_time	Time taken to verbal screen on reception into prison	Minutes	LN(2.271062,0.2510919)	6.3 (5.8-6.8)	Sutton et al. 2006 [21]
DurMDRTBfactor	Multiplier for MDR-TB treatment duration as a function of DS TB treatment duration	Ratio	B(69.7,162.6333)	0.3 (0.28 - 0.32)	Drobniewski et al. 2015 [22]
IncompTxfactor	Multiplier for non-completers treatment duration as a function of completers	Ratio	B(49.5,49.5)	0.3 (0.28 - 0.32)	Drobniewski et al. 2015 [22]
DurMDRTBIsolation	Duration of non-isolation inpatient (smear-negative MDR TB)	Days	LN(3.826432,0.06647485)	46 (44 - 48)	Drobniewski et al. 2015 [22]
DurMDRTB2Isolation	Duration of negative-pressure isolation (smear-positive MDR TB)	Days	LN(4.486828,0.0601382)	89 (85 - 92)	Drobniewski et al. 2015 [22]
DurDSTBIsolation	Duration of standard inpatient isolation	Days	LN(2.637757,0.05098725)	14 (13.46 - 14.48)	Drobniewski et al. 2015 [22]
pDSTB	Proportion of DSTB (Not resistant to any first line drug)	Proportion	B(461.2605,46.17679)	0.91 (0.90 - 0.92)	Calculated using data from TB diagnosis, microbiology and drug resistance in England, 2021 [19]
smear.positive	Proportion of sputum smear positive	Proportion	B(3242.953,1151.292)	0.738 (0.725 - 0.751)	Assumed based on Any test positive from TB diagnosis, microbiology and drug resistance in England, 2021 [19]
dstb.visits	Outpatient visits: DSTB	Visits	Fixed	6.1	Drobniewski et al. (2015) [22]
mdrtb.visits	Outpatient visits: MDRTB	Visits	Fixed	20.33	Drobniewski et al. (2015) [22]
DurDSTB	Treatment duration: DSTB (completers)	Days	Fixed	183	Drobniewski et al. (2015) [22]
DurTPT	Treatment duration: LTBI (completers)	Days	Fixed	90	Drobniewski et al. (2015) [22]
TPT.visits	Outpatient visits: LTBI	Visits	Fixed	3	Drobniewski et al. (2015) [22]

1.4.5 Unit costs

Table A13 Unit cost values and sources

Name	Description	Mean	SD	Source

cost.tb.sympt.screen	TB symptom screening prison/PPD nurse time	12.5	3.19	Calculated
cost.overheads	TB symptom screening space & materials	50	1.25	Assumed
cost.prison.cell.isolation	Prison single-cell or healthcare unit isolation	280.97	3.06	London Prison costings
cost.nhs.tb.service	NHS TB services outpatient visit	195	4.88	NHS reference costs
cost.prison.escort	Prison security & transport	122.78	1.28	London Prison costings
cost.tb.investigations	TB investigations	104.5	2.61	Drobniewski et al. (2015) [22]
cost.dstb.opd.visit	NHS TB services outpatient visit for DSTB	115.64	2.89	Drobniewski et al. (2015) [22]
cost.mdrtb.opd.visit	NHS TB services outpatient visit for MDRTB	171.87	4.30	Drobniewski et al. (2015) [22]
cost.dsatt.drugs	DSTB medicines	0.87	0.02	Drobniewski et al. (2015) [22]
cost.mdratt.drugs	MDR-TB medicines	21.2	0.53	Drobniewski et al. (2015) [22]
cost.att.dots	DOT costs Prison/PPD officer or nurse time	12.5	3.19	Calculated
cost.prison.gp.assess	Prison GP assessment	180	4.50	PSSRU Unit Costs in Criminal Justice[23]
cost.chest.xray	Chest X-rayÂ²	24.21	0.61	Drobniewski et al. (2015) [22]
cost.contact.tracing	Cost per person contact-traced	842	21.05	Drobniewski et al. (2015) [22]
cost.ltbi.drugs	LTBI medicines*	1.09	0.03	British National Formulary (BNF) [24]
cost.igra.test	IGRA test**	112	2.80	Abubakar et al. (2018) [25]
cost.mdrtb.ipd.smear.positive	Negative-pressure isolation (MDRTB)	1126	28.15	Drobniewski et al. (2015) [22]
cost.dstb.ipd	Standard isolation (DSTB)	390	9.75	Drobniewski et al. (2015)
cost.prison.bedwatch	Bedwatch	280.97	3.06	London Prison costings

* Daily Pyridoxine 10mg tablets and Rifampicin and Isoniazid 300mg/150mg tablets
** sum of QFT-GIT and T-SPOT.TB as recommended

1.5 Analytic approach

All results are based on probabilistic sensitivity analysis (PSA), sampled from all three parameter types: namely tree outputs determining outcomes and costs; TB natural history parameters; and parameters defining flows through the prison system. Results are based on samples of size 10,000. Samples from trees of the form summarized in Table 1 of the main paper and form the basis of the linkage between trees and the ODE-based model. Discrete sensitivity analyses were performed as described in 14. The model with prior input parameters predicted a TB notification rate among detainees of 123 (38 to 336) per 100,000 person-years, which compares with the estimate for the UK prison population of 144 (32 to 342) by Martinez et al.[26] One sensitivity analysis restricted to PSA samples with baseline TB notification rates among detainees between 30 and 100 per 100,000 per year. Interventions are modelled as instantaneously rolled out, and continuously-maintained (i.e. applied to all subsequent new receptions). We used Sheffield Accelerated Value of Information (SAVI)[27] to compute the per person expected value of partially perfect information (PPEVPPI) for each parameter.

1.6 Approach to modelling targeting

To represent a low-cost (modelled as no-cost) restriction of screening to a sub-population of those entering remand based on individual characteristics, we consider an additional pre-screening step ahead of that modelled with decision trees above. See 5. We consider this pre-screening step to have its own sensitivity and specificity (se_1 and sp_1) for identifying TB infection (assumed the same for both), that results in screening a fraction x of the pre-screening population, with prevalence of TB infection enriched by an odds ratio OR . Given the TB infection prevalence in the pre-screening population, the pre-screen sensitivity and specificity are in correspondence with x and $OR = odds\left(sp_1\right)/odds\left(se_1\right)$.

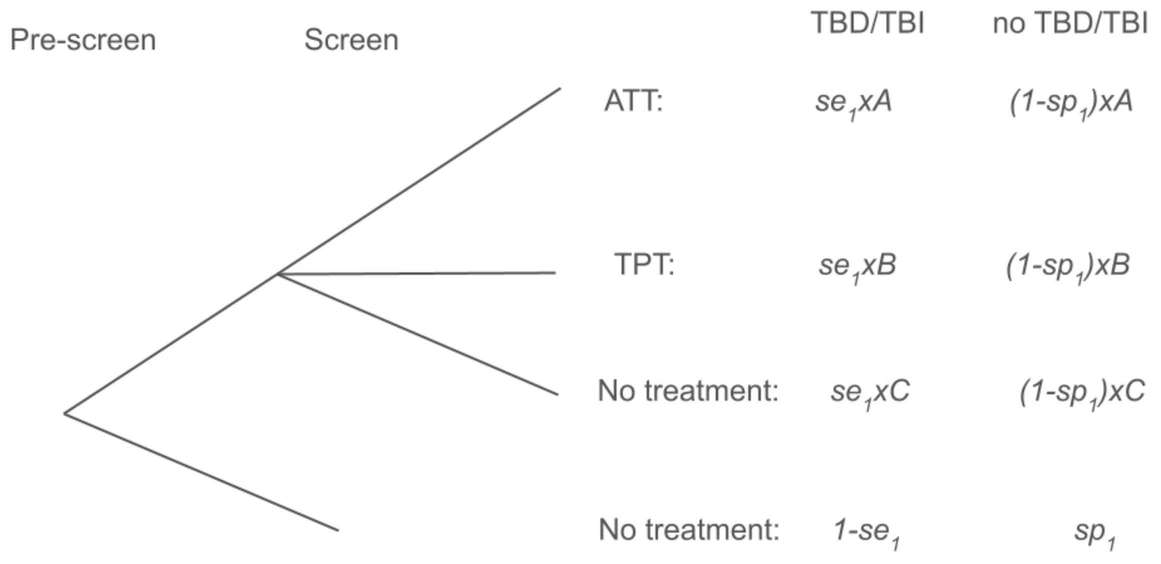

Figure A5 Structure of pre-screening step

Applying this pre-screen changes the (ATT,TPT, no treatment) X (TBD, TBI, no TB) matrix from:

A	a	α
B	b	β
C	c	γ

to

$se_1 A$	$se_1 a$	$(1 - sp_1)\alpha$
$se_1 B$	$se_1 b$	$(1 - sp_1)\beta$
$1 - se_1(1 - C)$	$1 - se_1(1 - c)$	$(1 - sp_1)\gamma + sp_1$

The commensurate matrix of unit costs U by outcome and disease state changes only the the bottom row

$\frac{se_1 C}{1-se_1(1-C)} \times U_{31}$	$\frac{se_1 c}{1-se_1(1-c)} \times U_{32}$	$\frac{(1-sp_1)\gamma}{(1-sp_1)\gamma+sp_1} \times U_{33}$

to account for changed denominators for those not receiving treatment. For fixed TB infection prevalence values, we present ICERs as two-dimensional results across implied values of x and OR.

1.7 Estimated sensitivity and specificity of targeting

With the notation:

x = proportion of population 'screened-in' (i.e. in target group)
L = TBI infection prevalence in whole population
L_1 = TBI infection prevalence in screened-in group
L_0 = TBI infection prevalence in screened-out group

we have:

se_1 = (screened-in positives)/(all positives) $= xL_1/L$
sp_1 = (screened-out negatives)/(all negatives) $= (1 - x)(1 - L_0)/(1 - L)$

Gray et al[28] found $L = 7\%$, which is consistent with data from the bio-behavioural survey (BBS). Analysis of BBS data and the prison population by country of birth yielded for group 1 (those born in countries with WHO-estimated TB incidence over 40/100,000 per year): $L_1 \approx 21\%$, $L_0 \approx 6\%$, and $x \approx 6\%$. Using the above relationships, this corresponds to $se_1 \approx 18\%$ and $sp_1 \approx 95\%$. For group 2 (the union of group 1 with those having a history of homelessness or injecting drug use), analysis of BBS data yielded $L_1 \approx 8\%$, and $L_0 \approx 5\%$. Available data on the prison population do not stratify by all 3 risk factors separately, but 15% of people in prison have a history of homelessness.[29,30] Accounting for overlaps in risk group indications, it was considered reasonable that $x = 20 - 40\%$ of the prison population would belong to group 2. Using the above formulae, this translates to ranges $se_1 = 23 - 46\%$ and $sp_1 = 61 - 83\%$. We therefore used $se_1 \approx 30\%$ and $sp_1 \approx 70\%$.
No other parameters were changed in defining targeted strategies.

2 Supplementary Results

Results from the SAVI analysis suggested that by far the most influential parameters for the decision with base case intervention were the parameters for fast (PPEVPI=£19 (SD=£4)) and slow progression (PPEVPI=£12 (SD=4)).

Table A14 ICERs for different sensitivity analysis. AttendNHS = all attend NHS referral, ContactTracing = removing contact tracing costs, DOTsCost = removing DOT costs, FUVisitsCost = removing treatment follow-up visits costs, InpatientCost = removing inpatient ATT costs, noltfu = no LTFU on (GP assessment, NHS referral, treatment initiation & completion), PrisonEscort = removing prison escort costs, XrayCost = removing X-ray costs.

analysis	ICER (per QALY gained), unrestricted TB rates	ICER (per QALY gained), restricted TB rates*
base case	£78,129	£168,226
Static model	£77,621	£167,370
Static model, no community transmission	£120,708	£253,595
Static model, no community transmission or post-TB effects	£170,465	£365,296
AllattendNHS	£77,621	£167,370
ContactTracing	£74,685	£160,741
DOTsCost	£68,950	£149,324
FUVisitsCost	£72,086	£156,273
InpatientCost	£64,134	£135,975
noltfu	£70,215	£152,731
PrisonEscort	£54,040	£118,051
XrayCost	£77,621	£167,370

* >30 & <100 per 100,000 notifications per person year

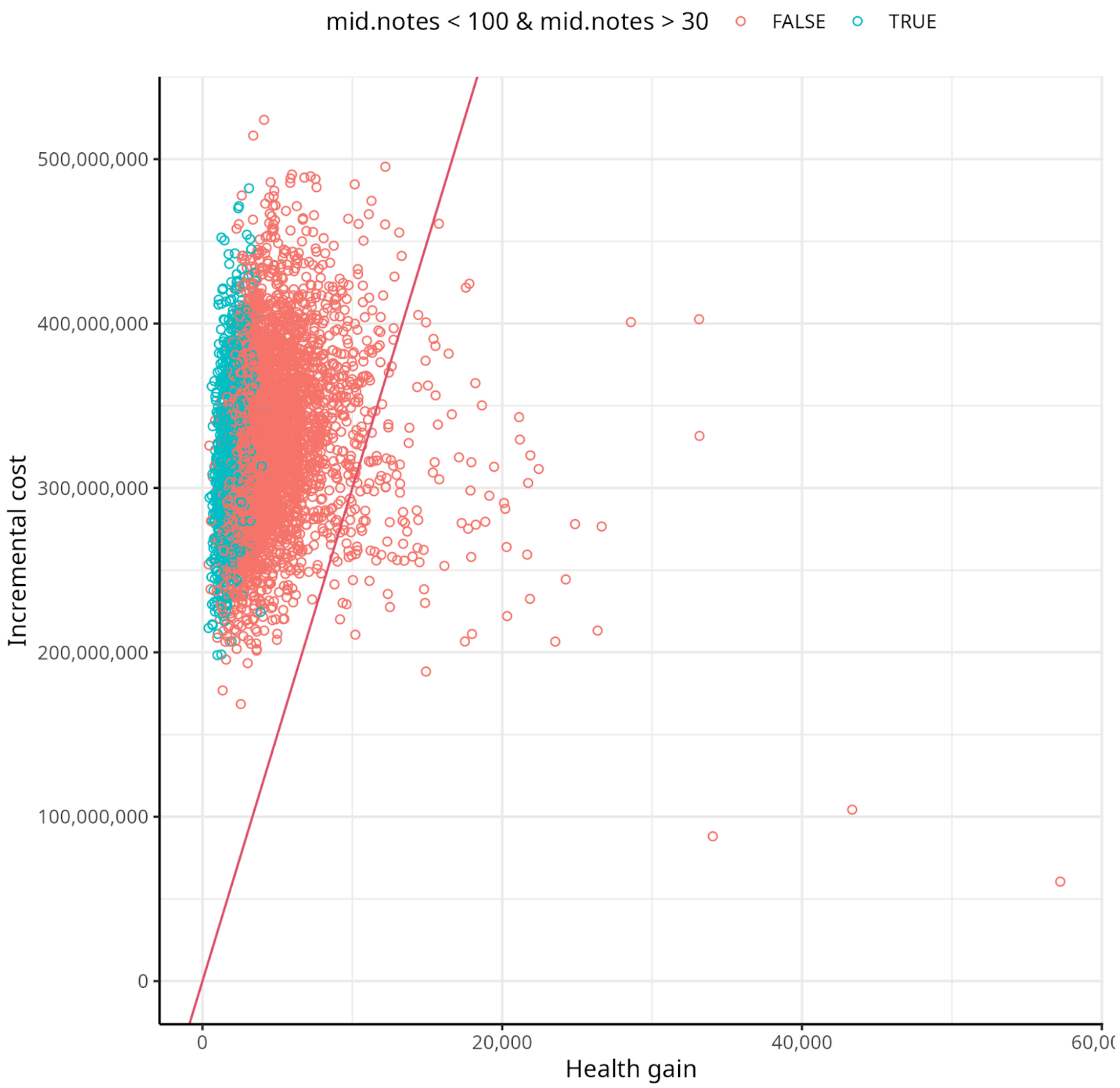

Figure A6 Cost-effectiveness plane showing incremental costs in GBP and QALYs gained, both discounted at 3.5%. The red line shows a cost-effectiveness threshold of £35 K per QALY gained.

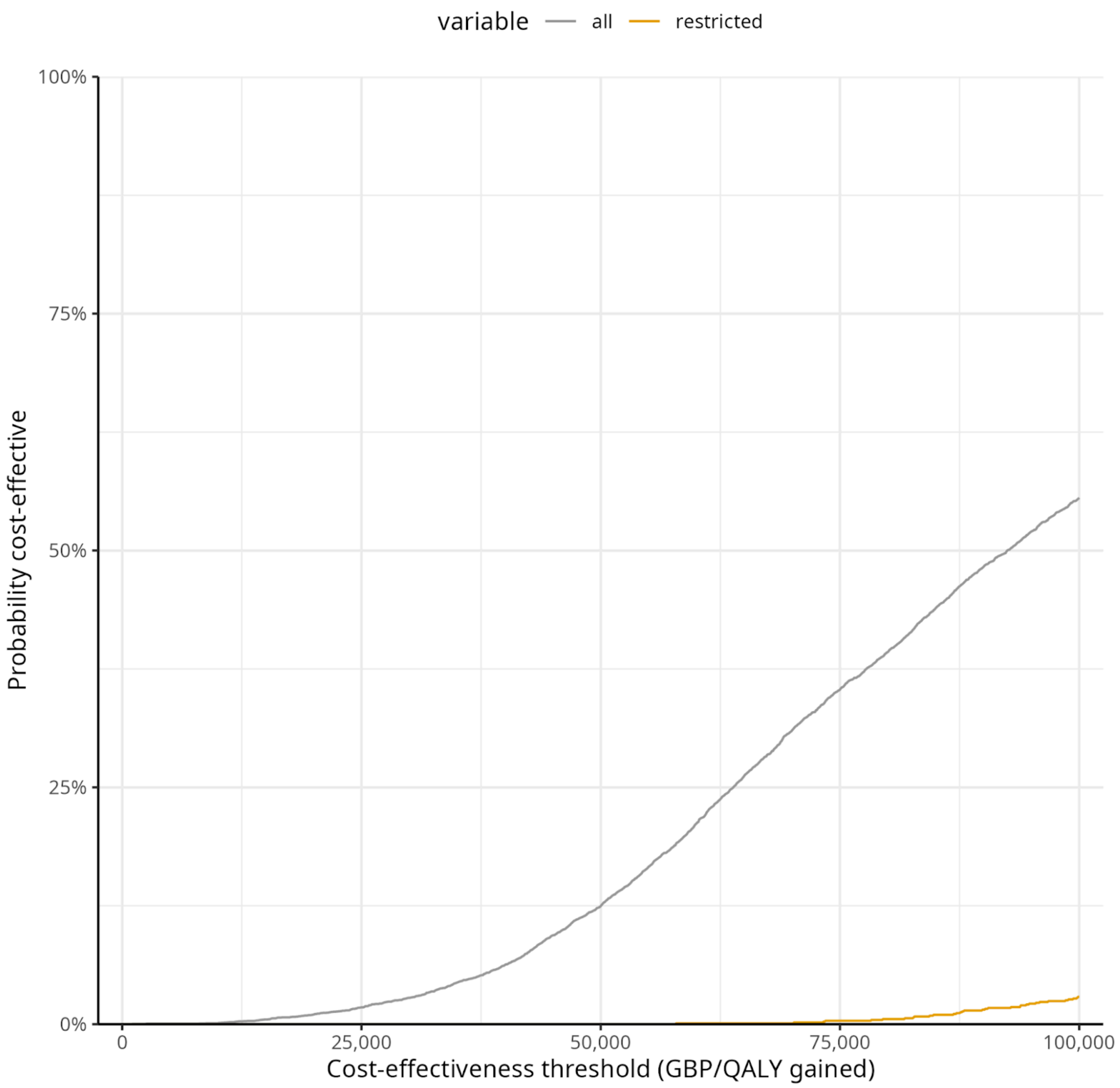

Figure A7 Cost-effectiveness acceptability curve showing the probability of being cost-effective at different cost-effectiveness thresholds in GBP per QALY gained. ‘Restricted’ refers to only outputs with TB notification rates in prisons between 30 and 100 per 100,000 person years.

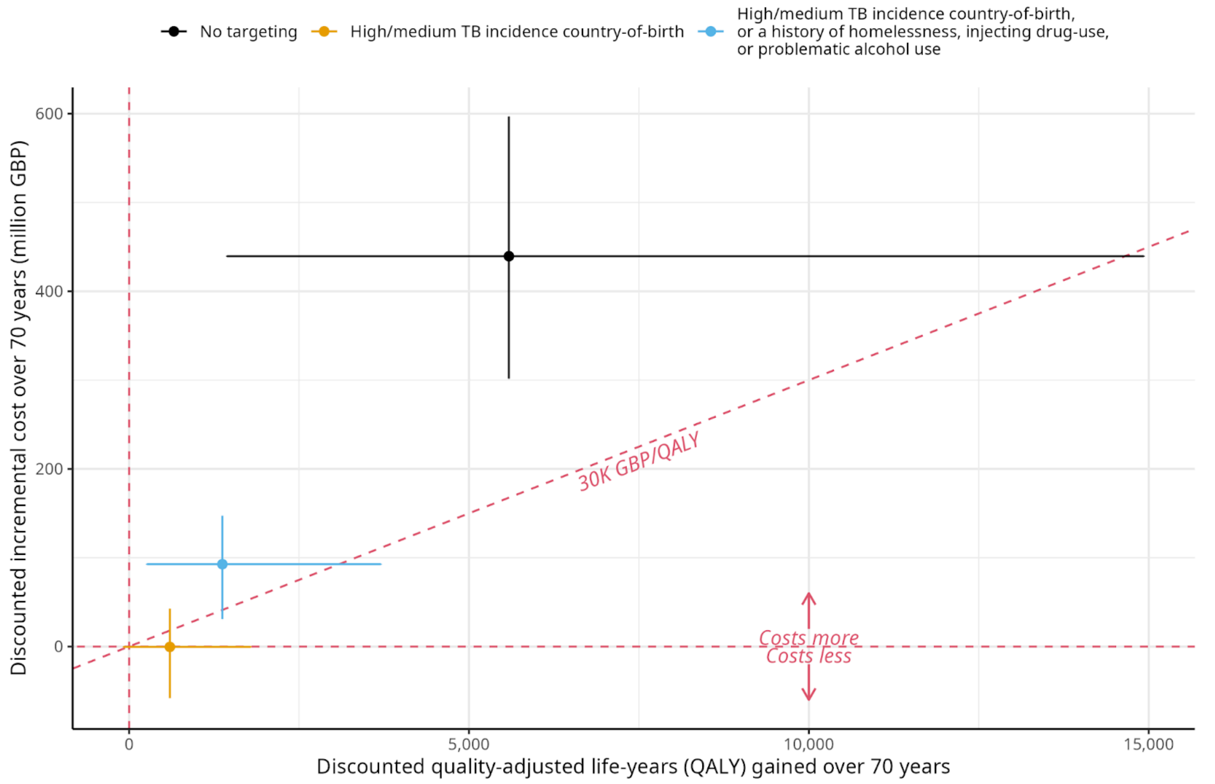

Figure A8 Cost-effectiveness plane for basecase and targeted interventions. The error bars denote the 95% uncertainty quantiles.

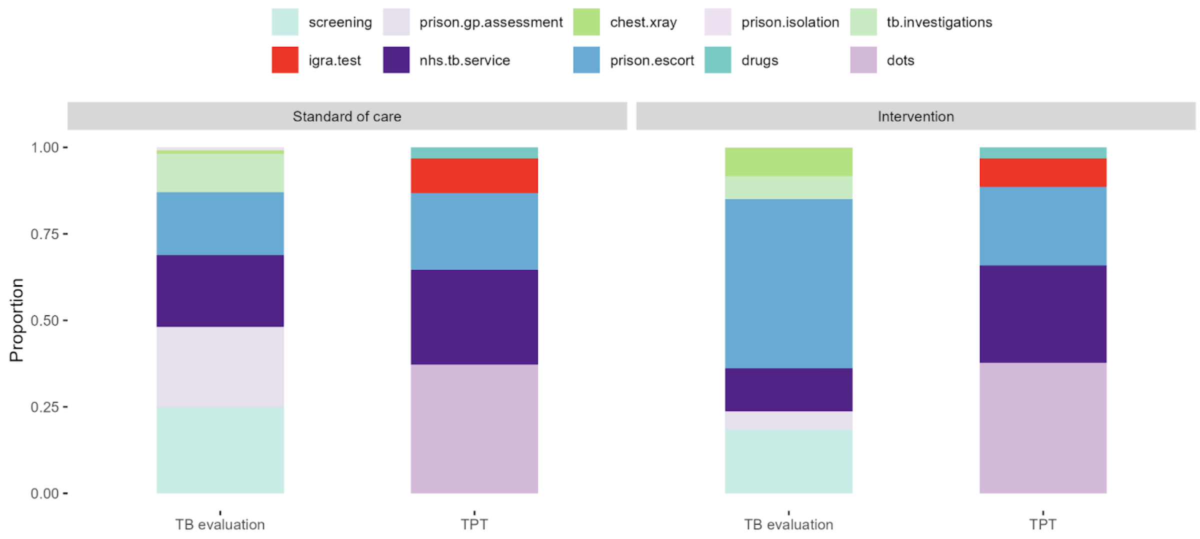

Figure A9 Breakdown of costs associated with tuberculosis preventive treatment (TPT)

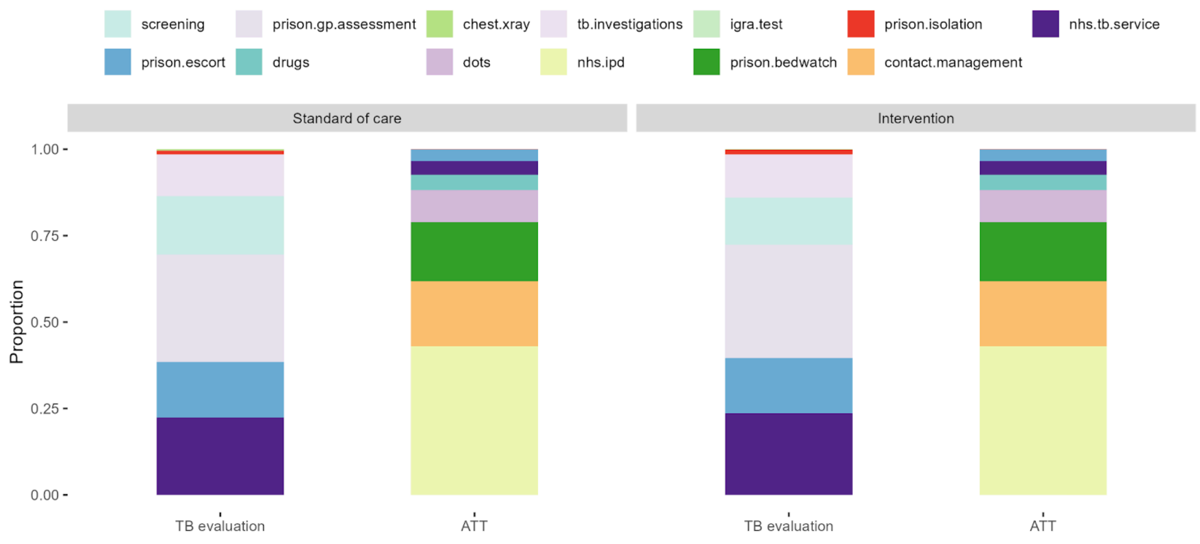

Figure A10 Breakdown of costs associated with anti-tuberculosis treatment (ATT)

3 References

1. Salazar-Austin N, Dowdy DW, Chaisson RE, Golub JE. Seventy years of tuberculosis prevention: Efficacy, effectiveness, toxicity, durability, and duration. Am J Epidemiol [Internet]. 2019 Dec 31 [cited 2024 Jul 3];188(12):2078–85. Available from: https://www.ncbi.nlm.nih.gov/pmc/articles/PMC7212403/

2. Emery JC, Dodd PJ, Banu S, Frascella B, Garden FL, Horton KC, Hossain S, Law I, van Leth F, Marks GB, Nguyen HB, Nguyen HV, Onozaki I, Quelapio MID, Richards AS, Shaikh N, Tiemersma EW, White RG, Zaman K, Cobelens F, Houben RMGJ. Estimating the contribution of subclinical tuberculosis disease to transmission: An individual patient data analysis from prevalence surveys. Elife [Internet]. 2023 Dec 18;12:e82469. Available from: http://dx.doi.org/10.7554/eLife.82469

3. Anderson, Moore, Kruijshaar, Pedrazzoli. Tuberculosis in the UK: Annual report on tuberculosis surveillance in the UK, 2010. London: Health Protection.

4. Glaziou P, Dodd PJ, Dean A, Floyd K. Methods used by WHO to estimate the global burden of TB disease. 2020; Available from: https://www.who.int/tb/publications/global_report/TB20_Technical_Appendix_20201014.pdf?ua=1

5. Tiemersma EW, van der Werf MJ, Borgdorff MW, Williams BG, Nagelkerke NJD. Natural history of tuberculosis: duration and fatality of untreated pulmonary tuberculosis in HIV negative patients: a systematic review. PLoS One [Internet]. 2011 Apr 4;6(4):e17601. Available from: http://dx.doi.org/10.1371/journal.pone.0017601

6. Tuberculosis (TB): action plan for England, 2021 to 2026 [Internet]. GOV.UK. [cited 2024 Jul 3]. Available from: https://www.gov.uk/government/publications/tuberculosis-tb-action-plan-for-england/tuberculosis-tb-action-plan-for-england-2021-to-2026

7. Ragonnet R, Trauer JM, Scott N, Meehan MT, Denholm JT, McBryde ES. Optimally capturing latency dynamics in models of tuberculosis transmission. Epidemics [Internet]. 2017 Jun 16;21:39–47. Available from: http://dx.doi.org/10.1016/j.epidem.2017.06.002

8. Andrews JR, Noubary F, Walensky RP, Cerda R, Losina E, Horsburgh CR. Risk of progression to active tuberculosis following reinfection with Mycobacterium tuberculosis. Clin Infect Dis [Internet]. 2012 Mar;54(6):784–91. Available from: http://dx.doi.org/10.1093/cid/cir951

9. Menzies NA, Quaife M, Allwood BW, Byrne AL, Coussens AK, Harries AD, Marx FM, Meghji J, Pedrazzoli D, Salomon JA, Sweeney S, van Kampen SC, Wallis RS, Houben RMGJ, Cohen T. Lifetime burden of disease due to incident tuberculosis: a global reappraisal including post-tuberculosis sequelae. Lancet Glob Health [Internet]. 2021 Dec 1;9(12):e1679–87. Available from: https://www.thelancet.com/journals/langlo/article/PIIS2214-109X(21)00367-3/fulltext?s=09#

10. Crampin AC, Mwaungulu JN, Mwaungulu FD, Mwafulirwa DT, Munthali K, Floyd S, Fine PE, Glynn JR. Recurrent TB: relapse or reinfection? The effect of HIV in a general population cohort in Malawi. AIDS [Internet]. 2010 Jan 28;24(3):417–26. Available from: http://dx.doi.org/10.1097/QAD.0b013e32832f51cf

11. Frascella B, Richards AS, Sossen B, Emery JC, Odone A, Law I, Onozaki I, Esmail H, Houben RMGJ. Subclinical tuberculosis disease - a review and analysis of prevalence surveys to inform definitions, burden, associations and screening methodology. Clin Infect Dis [Internet]. 2020 Sep 16; Available from: http://dx.doi.org/10.1093/cid/ciaa1402

12. Martinez L, Seddon JA, Horsburgh CR, Lange C, Mandalakas AM, TB Contact Studies Consortium. Effectiveness of preventive treatment among different age groups and Mycobacterium tuberculosis infection status: a systematic review and individual-participant data meta-analysis of contact tracing studies. Lancet Respir Med [Internet]. 2024 May 8; Available from: http://dx.doi.org/10.1016/S2213-2600(24)00083-3

13. Romanowski K, Baumann B, Basham CA, Ahmad Khan F, Fox GJ, Johnston JC. Long-term all-cause mortality in people treated for tuberculosis: a systematic review and meta-analysis. Lancet Infect Dis [Internet]. 2019 Oct;19(10):1129–37. Available from: http://dx.doi.org/10.1016/S1473-3099(19)30309-3

14. Romanowski K, Law MR, Karim ME, Campbell JR, Hossain MB, Gilbert M, Cook VJ, Johnston JC. Healthcare Utilization After Respiratory Tuberculosis: A Controlled Interrupted Time Series Analysis. Clin Infect Dis [Internet]. 2023 Sep 18;77(6):883–91. Available from: http://dx.doi.org/10.1093/cid/ciad290

15. Cords O, Martinez L, Warren JL, O'Marr JM, Walter KS, Cohen T, Zheng J, Ko AI, Croda J, Andrews JR. Incidence and prevalence of tuberculosis in incarcerated populations: a systematic review and meta-analysis. The Lancet Public Health [Internet]. 2021 Mar 22; Available from: https://www.sciencedirect.com/science/article/pii/S2468266721000256

16. World Health Organization. WHO consolidated guidelines on tuberculosis: Module 2: screening – systematic screening for tuberculosis disease [Internet]. Geneva: World Health Organization; 2021. Available from: https://www.who.int/publications/i/item/9789240022676

17. Zifodya JS, Kreniske JS, Schiller I, Kohli M, Dendukuri N, Schumacher SG, Ochodo EA, Haraka F, Zwerling AA, Pai M, Steingart KR, Horne DJ. Xpert Ultra versus Xpert MTB/RIF for pulmonary tuberculosis and rifampicin resistance in adults with presumptive pulmonary tuberculosis. Cochrane Database Syst Rev [Internet]. 2021 Feb 22 [cited 2024 Jul 9];2(5):CD009593. Available from: https://pubmed.ncbi.nlm.nih.gov/33616229/

18. Davies M, Rolewicz L, Schlepper L, Fagunwa F. Locked out? Prisoners' use of hospital care. Nuffield Trust; 2020.

19. TB treatment outcomes in England, 2021 [Internet]. GOV.UK. [cited 2024 Jul 9]. Available from: https://www.gov.uk/government/publications/tuberculosis-in-england-2022-report-data-up-to-end-of-2021/tb-treatment-outcomes-in-england-2021

20. Pareek M, Bond M, Shorey J, Seneviratne S, Guy M, White P, Lalvani A, Kon OM. Community-based evaluation of immigrant tuberculosis screening using interferon γ release assays and tuberculin skin testing: observational study and economic analysis. Thorax [Internet]. 2013 Mar [cited 2024 Jul 9];68(3):230–9. Available from: https://pubmed.ncbi.nlm.nih.gov/22693179/

21. Sutton AJ, Edmunds WJ, Gill ON. Estimating the cost-effectiveness of detecting cases of chronic hepatitis C infection on reception into prison. BMC Public Health [Internet]. 2006 Jun 27 [cited 2024 Jul 9];6(1):170. Available from: https://bmcpublichealth.biomedcentral.com/articles/10.1186/1471-2458-6-170

22. Drobniewski F, Cooke M, Jordan J, Casali N, Mugwagwa T, Broda A, Townsend C, Sivaramakrishnan A, Green N, Jit M, Lipman M, Lord J, White PJ, Abubakar I. Systematic

review, meta-analysis and economic modelling of molecular diagnostic tests for antibiotic resistance in tuberculosis. Health Technol Assess [Internet]. 2015 May;19(34):1–188, vii – viii. Available from: http://dx.doi.org/10.3310/hta19340

23. Brookes N, Barrett B, Netten A. Unit Costs in Criminal Justice (UCCJ).

24. Medicinal forms [Internet]. [cited 2024 Jul 9]. Available from: https://bnf.nice.org.uk/drugs/rifampicin-with-isoniazid/medicinal-forms/

25. Abubakar I, Lalvani A, Southern J, Sitch A, Jackson C, Onyimadu O, Lipman M, Deeks JJ, Griffiths C, Bothamley G, Kon OM, Hayward A, Lord J, Drobniewski F. Two interferon gamma release assays for predicting active tuberculosis: the UK PREDICT TB prognostic test study. Health Technol Assess [Internet]. 2018 Oct [cited 2024 Jul 9];22(56):1–96. Available from: https://www.ncbi.nlm.nih.gov/books/NBK532121/

26. Martinez L, Warren JL, Harries AD, Croda J, Espinal MA, Olarte RAL, Avedillo P, Lienhardt C, Bhatia V, Liu Q, Chakaya J, Denholm JT, Lin Y, Kawatsu L, Zhu L, Horsburgh CR, Cohen T, Andrews JR. Global, regional, and national estimates of tuberculosis incidence and case detection among incarcerated individuals from 2000 to 2019: a systematic analysis. The Lancet Public Health [Internet]. 2023 Jul 1;8(7):e511–9. Available from: https://doi.org/10.1016/S2468-2667(23)00097-X

27. Strong M, Oakley JE, Brennan A. Estimating Multiparameter Partial Expected Value of Perfect Information from a Probabilistic Sensitivity Analysis Sample A Nonparametric Regression Approach. Med Decis Making [Internet]. 2014 Apr 1;34(3):311–26. Available from: http://mdm.sagepub.com/content/34/3/311

28. Gray BJ, Perrett SE, Gudgeon B, Shankar AG. Investigating the prevalence of latent Tuberculosis infection in a UK remand prison. J Public Health (Oxf) [Internet]. 2020 Feb 28 [cited 2024 Jul 17];42(1):e12–7. Available from: https://academic.oup.com/jpubhealth/article-pdf/42/1/e12/32650848/fdy219.pdf

29. Prison Reform Trust. January 2023 Bromley Briefings [Internet]. 2023. Available from: https://prisonreformtrust.org.uk/wp-content/uploads/2023/02/January-2023-Bromley-Briefings.pdf

30. Alcohol and drug treatment in secure settings 2022 to 2023: report [Internet]. GOV.UK. [cited 2024 Dec 18]. Available from: https://www.gov.uk/government/statistics/substance-misuse-treatment-in-secure-settings-2022-to-2023/alcohol-and-drug-treatment-in-secure-settings-2022-to-2023-report--2
